# IL-18 and IL-18 binding protein are related to disease severity and parasitemia during falciparum malaria

**DOI:** 10.1186/s12879-021-06751-y

**Published:** 2021-10-18

**Authors:** Kari Otterdal, Aase Berg, Annika E. Michelsen, Arne Yndestad, Sam Patel, Ida Gregersen, Bente Halvorsen, Thor Ueland, Nina Langeland, Pål Aukrust

**Affiliations:** 1 Research Institute of Internal Medicine, Oslo University Hospital Rikshospitalet, Nydalen, PO Box 4950, 0424 Oslo, Norway; 2 Department of Medicine, Stavanger University Hospital, PO Box 8100, 4068 Stavanger, Norway; 3 Department of Medicine, Central Hospital of Maputo, Maputo, Mozambique; 4 Faculty of Medicine, University of Oslo, 0316 Oslo, Norway; 5 K.G. Jebsen Thrombosis Research and Expertise Center, University of Tromsø, 9019 Tromsø, Norway; 6 Department of Clinical Science, University of Bergen, 5021 Bergen, Norway; 7 Department of Medicine, Haukeland University Hospital, 5021 Bergen, Norway; 8 Department of Medicine, Haraldsplass Deaconess Hospital, 5009 Bergen, Norway; 9 Section of Clinical Immunology and Infectious Diseases, Oslo University Hospital Rikshospitalet, 0372 Oslo, Norway

**Keywords:** IL-18, IL-18bp, Falciparum malaria, HIV, Endothelial cells

## Abstract

**Background:**

Several inflammatory molecules participate in the immune response to malaria. Interleukin (IL)-18 is an inflammatory cytokine activated by NLRP3 inflammasomes. In clinical falciparum malaria, with and without HIV co-infection, data on IL-18 and in particular on its binding protein, IL-18bp, is scarce.

**Methods:**

Clinical data and blood samples were collected from adults in Mozambique with *P. falciparum* infection, with (n = 70) and without (n = 61) HIV co-infection, from HIV-infected patients with similar symptoms without malaria (n = 58) and from healthy controls (n = 52). In vitro studies were performed in endothelial cells using hemozoin crystals.

**Results:**

(i) IL-18 and IL-18bp were markedly up-regulated during falciparum malaria with particular high levels in malaria patients co-infected with HIV and severe malaria disease. (ii) In the malaria group as a whole, both IL-18 and IL-18bp were positively correlated with disease severity, parasitemia, and endothelial cell activation as assessed by vWF in plasma. (iii) Whereas there was no change in IL-18 levels in malaria patients co-infected with HIV during follow-up, the patients with malaria only had slightly increased IL-18 levels. Further, the IL-18pb levels declined and thereby contributed to an increase in IL-18/IL-18bp ratio in all subgroups of malaria patients. (iv) IL-27, previously shown to be up-regulated in this malaria cohort, markedly induced a release of IL-18bp from endothelial cells in vitro, and notably, this presumably anti-inflammatory effect was counteracted by hemozoin.

**Conclusions:**

Our findings suggest that the IL-18 system could be an important mediator in the immune pathogenesis during falciparum malaria, potentially also representing a target for therapy***.***

## Introduction

*Plasmodium falciparum* (*P. falciparum*) malaria is an important cause of morbidity and mortality in sub-Saharan Africa. The interaction between the parasite and the immune system has been extensively examined both in experimental and clinical studies. These data illustrate that the balance between a well-adjusted and an overwhelming immune response is critical for the outcome of falciparum malaria, mediating beneficial and harmful responses, respectively, in the infected host [1, 2].

Interleukin (IL)-18 is an inflammatory cytokine, activated by NLRP3 inflammasomes, and released from the cell promoting inflammatory responses (e.g., induction of interferon (IFN) γ release) in T cells and natural killer (NK) cells. The biological effects of IL-18 are augmented by the IL-18 binding protein (IL-18bp) which acts as a soluble decoy receptor and circulates at > tenfold higher level than IL-18. IL-18 binds to IL-18bp with an affinity significantly higher than that of IL-18 receptor α [3, 4]. Whereas IL-18 may have protective effects in various models of experimental malaria [5], high levels of IL-18 have been associated with disease severity in clinical *P. falciparum* infection [6–8]. However, few studies, with a relative low number of patients, have examined circulating IL-18 levels in falciparum malaria [6–8]. Moreover, whereas we and others have shown that HIV infection is associated with increased IL-18 levels [9, 10], there are no data on how HIV infection influences IL-18 levels in patients with *P. falciparum* infection. Furthermore, except for a large study in pregnant women [11], there are no data on IL-18bp in patients with falciparum malaria.

In the present study we examined plasma levels of IL-18 and IL-18bp in a cohort of non-pregnant adult patients with falciparum malaria with and without HIV co-infection, arriving at the Central Hospital of Maputo, Mozambique. IL-18 and IL-18bp levels were related to disease severity and parasitemia as assessed by quantitative *P. falciparum* PCR analyses. We also examined the ability of the malarial pigment hemozoin to modulate the release of IL-18 and IL-18bp from endothelial cells. This was performed in the presence of IL-27, previously shown to be up-regulated in this malaria cohort [12]. We have also recently shown that IL-27 can promote NLRP3 activation, which is an important source for IL-18 [13]. In addition, others have shown synergistic effect of IL-27 with IL-18 in NK cells [14] and IL-27 dependent up-regulation of IL-18bp in human ovarian cancer cells and skin resident cells [15, 16], suggesting some interactions between IL-27 and IL-18 during inflammation and immune responses.

## Materials and methods

### Description of study design and participants

The study design has previously been described [17]. Briefly, in this prospective, cross-sectional study, we included 212 patients admitted to the Medical Emergency Department in the Central Hospital of Maputo, Mozambique during seven months in two malaria peak seasons, from 2011 to 2012. Inclusion criteria were age ≥ 18 years, non-pregnancy, axillary temperature ≥ 38 °C and/or clinical suspected or confirmed malaria infection, and consent from patient or next of kin. Of the 212 screened patients, 129 had *P. falciparum* malaria as assessed by qualitative PCR, one by quantitative PCR and one that had no PCR done had positive RDT and thick slide, giving a total of 131 falciparum malaria patients. Of the malaria patients, the median age was 37 years (18–84 years), 47% were women, and 53% were co-infected with HIV-1 as assessed by PCR and/or serological tests. Of the malaria patients, 92% received quinine intravenously as a first-line therapy, 4% received artemether intramuscularly, and the rest were treated with oral artemisinin combinations [17].

Severe malaria was found in 65% (85/131) of the patients, which meant that the patients had at least one of the WHO severity criteria [17, 18]. The severity criteria were: repeated convulsions and/or impaired consciousness with Glasgow Coma Score < 11, severe hypoglycaemia with plasma glucose < 2.2 mmol/L, severe anemia with Hb < 5 g/dL, renal failure with creatinine > 265 µmol/L, liver failure with bilirubin > 50 µmol/L, signs of respiratory failure with pulmonary oedema on x-rays and/or respiratory rate > 30/minute, bleeding or signs of coagulopathy, hyperparasitemia > 4%, hyperthermia with temperature > 40 °C or signs of circulatory collapse/shock with systolic BP < 70 mmHg. 7.6% of the malaria patients died (10/128 of which nine were co-infected with HIV; missing data on outcome in three patients). The characteristics of the patient groups at admission are shown in Table 1. The qualitative *P. falciparum* PCR in whole blood and the quantitative *P. falciparum* PCR in plasma were performed as previously described [19, 20].

**Table 1:** Clinical characteristics of the patient population at admission

Characteristic	Malaria	HIV	Malaria + HIV
N	61	58	70
Age, years	40 (18–79)	39 (22–84)	40 (20–65)
Sex, females (% (n))	41 (25)	50 (29)	50 (35)
Haemoglobin (g/dL)	11.2 (3.2–17.0)	8.9 (2.9–15.2)	9.4 (2.5–15.7)
Leukocytes (× 10^9^/L)	6.9 (1.3–15.5)	8.2 (0.3–25.4)	7.8 (0.9–21.8)
Platelets (× 10^9^/L)	124 (11–452)	220 (13–682)	90 (8–330)
Se-Creatinine (µmol/L)	127 (57–357)	161 (41–873)	223 (62–1529)
Se-Glucose (mmol/L)	8.7 (3.6–40.5)	6.1 (3.3–10.6)	6.12 (1.5–27.0)
Liver failure (%)^a^	5 (3/61)	7 (4/57)	17 (12/70)
Coagulation disturbance (%)^b^	2 (1/61)	0	13 (9/70)
Cerebral affection (%)^c^	25 (15/61)	33 (19/58)	31 (22/70)
Systolic blood pressure	122 (70–240)	115 (90–160)	115 (80–170)
Respiratory rate	22 (12–68)	29 (12–56)	24 (16–42)
Case fatality rate (%)	1.7 (1/59)	27.8 (15/54)	13.0^d^ (9/69)
Duration of symptoms in days (median)	4.2 (1–28)	7 (1–365)	8.6 (1–180)
Severe HIV^e^ (%)	n.a	83 (48/58)	59 (41/70)
HIV viral load in copies/mL (median)	n.a	1.3 × 10^4^	1.8 × 10^4^
Median CD4 lymphocyte count (cells/μL)^f^	n.a	136	206
Effective ART^g^ prior to admission (%)	n.a	19 (10/53)	14 (9/64)

Values in mean (min–max) or percentage and proportion. The 52 healthy controls are not included ^a^Defined as jaundice/bilirubine > 50 µmol/L, ^b^Defined as bleeding disturbances/hemolysis, ^c^Defined as GCS ≤ 11, convulsions or confusion, ^d^One patient died of non-malarial cause, he was excluded, ^e^Severe HIV = WHO stage 3 or 4, ^f^CD4 T-cell count were only obtained in 8 (HIV only) and 11 (HIV + malaria) patients, ^g^ART = antiretroviral therapy = HIV treatment. ‘‘Effective’’ is defined as ‘‘Previous known ART and undetectable HIV-RNA in the plasma’’, in relation to all HIV-patients with and without malaria

For comparison, we also included 58 HIV-1-infected patients where malaria was excluded but with similar symptoms as the patients with falciparum malaria (fever, chills, headache, mental confusion, dyspnea, vomiting and/or diarrhea, myalgia and/or general malaise) and with similar HIV-related parameters as those co-infected with malaria (Table 1). These patients were diagnosed with, among others, tuberculosis, bacterial pneumonia, viral hepatitis, *pneumocystis jeroveci* pneumonia, toxoplasmosis encephalitis, urinary tract infection, and sepsis. Fifty-two apparently healthy HIV negative and malaria negative volunteers with median age 29 years (18–56 years, 40% women), were enrolled from hospital employees, and provided no history of chronic disease, had a subjective feeling of wellbeing and a healthy appearance evaluated by the researchers.

### Blood sampling protocol

Blood samples from patients and healthy controls were collected from a peripheral vein into EDTA-tubes on admission and after 48 h. The tubes were immediately placed on melting ice and centrifuged within 30 min at 2000*g* for 20 min to obtain platelet-poor plasma. Plasma was aliquoted and stored at − 20 °C for 24 h; then at − 80 °C.

### Endothelial cell culture

Primary Human Aortic Endothelial cells (HAoECs) were obtained from PromoCell GmbH, Heidelberg, Germany. The cells were cultured in Endothelial Cell Growth Medium MV2 (PromoCell), passaged by treatment with Trypsin/EDTA (0.04%/0.03%) and grown in 48-well plates (Thermo Scientific, Roskilde, Denmark) coated with 1% gelatin (Sigma, St Louis, MO). In the experiments the cells were cultured with and without recombinant human (rh)IL-27 (100 ng/mL; R&D Systems, Minneapolis, MN) in Opti-MEM reduced serum medium (Gibco, Grand Island, NY) supplemented with 5% FBS for 1 h before stimulated with different concentrations of chemically synthesized hemozoin (Invivogen, San Diego, CA) for 22 h. For evaluation of possible cell toxicity different concentration of hemozoin was tested in HAoEC cultures using Cytotoxicity Detection Kit from Sigma Aldrich (St. Louis, MO).

### Supernatant and plasma analyses

Protein levels of IL-18 and IL-18bp in plasma and cell supernatants were measured by enzyme immunoassays (EIAs) from R&D Systems. The intra- and interassay coefficient of variation were < 10% for all assays. Plasma levels of von Willebrand factor (vWF) have previously been analysed and reported in this population [12] and now used in correlation analyses.

### Real-time quantitative RT-PCR

Total RNA was obtained from HAoEC by using RNeasy spin columns (QIAGEN, Hilden, Germany). Samples were subjected to DNase treatment (RNase-Free DNase Set; QIAGEN) and stored at -80 °C until further analysis. cDNA synthesis was performed using qScript cDNA Supermix (Quanta Bio, Beverly, MA 01,915). Gene expression was examined by real-time quantitative (q)PCR. mRNA detection of IL-18 and IL-18bp and the reference gene β-actin was assessed with SybrGreen primers (Sigma Aldrich, St. Louis, MO 63103): IL-18, forward primers (FP): TCTTCATTGACCAAGGAAATCGG, reverse primers (RP): TCCGGGGTGCATTATCTCTAC; IL-18bp, FP: TGGAAGTGCCACTGAATGGA, RP: CCATTGCCCAGCCAGTAGAG; β-actin, FP: AGGCACCAGGGCGTGAT, RP: TCGTCCCAGTTGGTGACGAT. The relative mRNA level of each transcript was calculated by the ΔΔCt-method and normalized to controls.

### Statistical analyses

The distribution of IL-27 and vWF was skewed and nonparametric statistics were used throughout. For comparison between the diagnostic groups, Kruskal Wallis was used a priori followed by Dunn’s multiple comparison test between individual groups. Wilcoxon matched-pairs signed rank test was used to compare changes from baseline to follow-up within each diagnostic group. Spearman correlation was used to assess associations between variables. In the ex vivo experiments Student’s t test was used. A two-sided p < 0.05 was considered significant.

## Results

### IL-18, IL-18bp and their ratio in *P. falciparum* infection with and without HIV infection

The patient population is described in Table 1, consisting of three groups of patients: (i) HIV-infected patients with similar symptoms as malaria patients but with negative test for *P. falciparum* infection (n = 58), (ii) malaria patients without HIV infection (n = 61) and (iii) malaria patients co-infected with HIV (n = 70).

**Fig. 1:**
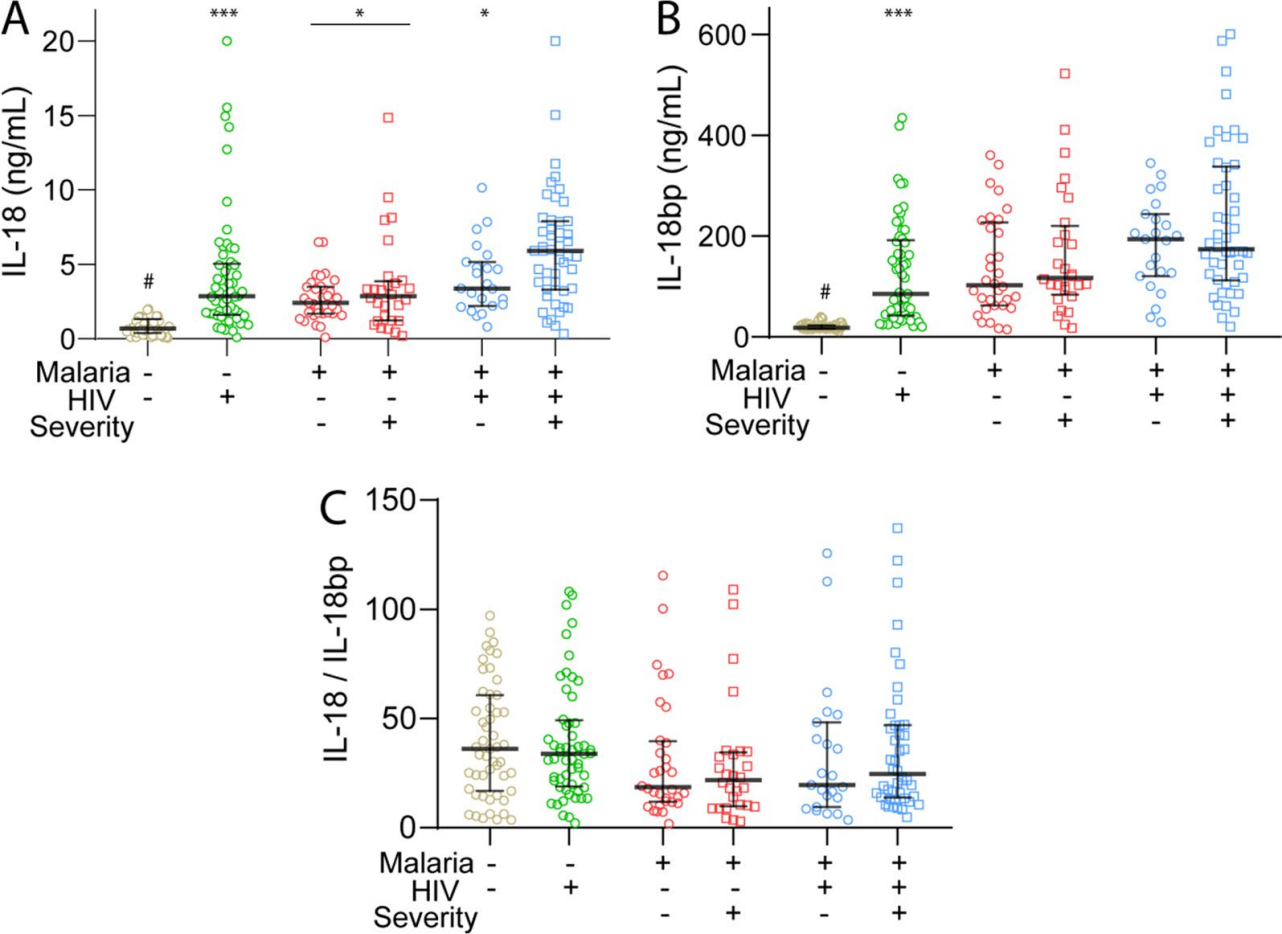
Plasma levels of IL-18 (**A**), IL-18bp (**B**) and the **C** IL-18/IL-18bp ratio according to severity. IL-18 and IL-18bp were measured in plasma in patients with HIV infection with febrile symptoms but without malaria (n = 58), patients with falciparum malaria without (M, n = 61 of which 28 with severe disease) and with HIV infection (HIV + M, n = 70 of which 47 with severe disease). For comparison, data from healthy controls (CTR, n = 52) are also included. Lines in the scatter plot represent the median and 25–75th percentiles. ^#^p < 0.001 vs. all patient groups; *p < 0.05, ***p < 0.001 versus HIV + M with severe malaria. In **A** the line with * indicates the Malaria without HIV group as a whole

There was a marked increase in plasma levels of IL-18 in all three patient groups compared to healthy controls (Fig. 1A). Interestingly, however, whereas there were no significant differences between those with malaria only comparing severe and mild disease, patients with malaria co-infected with HIV had significantly raised IL-18 levels compared not only to those with combined infection and mild disease, but also to all other subgroups of patients including those with malaria only and severe disease (Fig. 1A).

IL-18 bioactivity is attenuated by IL-18bp by preventing the binding of IL-18 to its receptor [21]. Interestingly, plasma levels of IL-18bp showed a similar pattern as IL-18 with a marked increase in all the three patient groups compared to healthy controls with the highest levels in the malaria patients co-infected with HIV and severe disease (Fig. 1B). In fact, these patients, but not co-infected patients with mild malaria disease, showed increased levels of IL-18bp also as compared with HIV-infected patients without malaria (Fig. 1B). The IL-18/IL-18bp ratio could potentially represent an estimate on IL-18 bioactivity. As depicted in Fig. 1C, this ratio showed no differences between the different malaria and severity groups, even as compared with healthy controls, potentially reflecting a rise in both IL-18 and IL-18bp in the patient groups.

### Associations of IL-18 and IL-18bp with clinical and biochemical characteristics in patients with P. falciparum infection with and without HIV infection

**Fig. 2:**
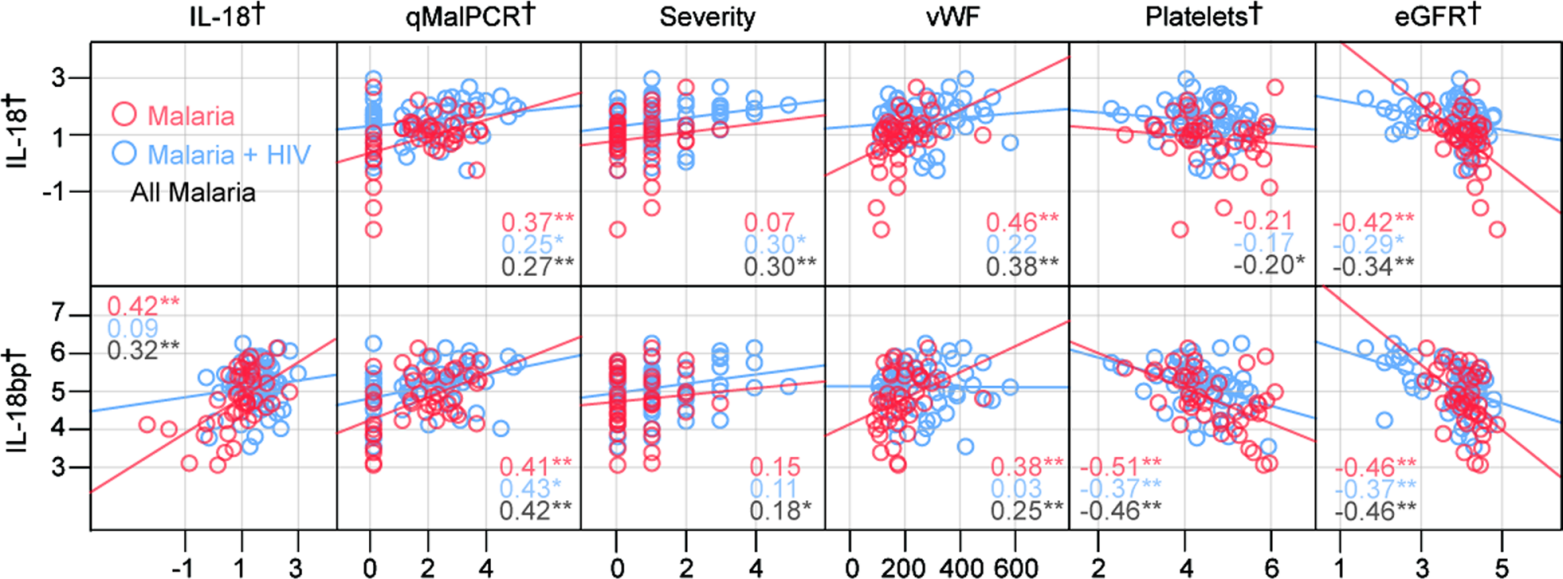
Scatterplots showing correlations (Spearman) between IL-18, IL-18bp and different parameters. Associations with parasitemia (qMalPCR), disease severity (the disease severity score was based on the WHO definition for malaria severity, see methods), plasma vWF, platelet counts and kidney function (eGFR) is shown. †log transformed for presentation. Numbers in graphs represent the correlation coefficient (rho) with red representing malaria, blue representing malaria + HIV and black all malaria patients. *p < 0.05, **p < 0.01

In malaria patients as a whole, IL-18 correlated positively with degree of parasitemia (assessed by qPCR), disease severity (defined by the WHO criteria [18]) and degree of endothelial cell activation (assessed by plasma vWF levels) and negatively with haemoglobin levels, platelet counts and kidney function (assessed by eGFR) (Table 2 and Fig. 2). Regarding parasitemia and kidney function, a similar pattern was seen in both the two malaria groups. Association with disease severity and vWF, this was found in malaria patients co-infected with HIV and in malaria patients only, respectively. No correlations with haemoglobin levels or platelet counts was found in the two groups of malaria patients with or without HIV (Table 2). Moreover, in the malaria patients as a whole IL-18bp correlated positively with degree of parasitemia, disease severity, IL-18 levels and plasma vWF and negatively with platelet counts and kidney function, but in contrast to IL-18, IL-18bp levels were not correlated with haemoglobin levels except from a positive association in malaria patients co-infected with HIV (Table 2). Moreover, whereas IL-18 and IL-18bp were significantly correlated in patients with malaria without accompanying HIV infection, this was not seen in malaria patients co-infected with HIV (Table 2). The most important correlations for IL-18 and IL-18bp are also shown in Fig. 2.

**Table 2:** Correlation between IL-18 and IL-18bp and clinical data in patients

	IL-18			IL-18bp		
	M	M + HIV	All	M	M + HIV	All
IL-18bp	0.42**	0.09	0.32**	–	–	–
qMalPCR	0.37**	0.25*	0.27**	0.41**	0.43**	0.42**
eGFR	− 0.42**	− 0.29*	− 0.34**	− 0.46**	− 0.34**	− 0.38**
Haemoglobin	− 0.07	− 0.22	− 0.22*	0.18	0.26*	0.12
Platelets	− 0.21	− 0.17	− 0.20*	− 0.51**	− 0.37**	− 0.46**
Neutrophils	− 0.13	− 0.30*	− 0.26*	0.23	− 0.07	0.03
Lymphocytes	0.10	0.32	0.25*	− 0.36*	0.01	− 0.12
WBC	0.29*	0.07	0.18	0.28*	0.03	0.14
vWF	0.46**	0.22	0.38**	0.38**	0.03	0.25**
Severity	0.07	0.30*	0.30**	0.15	0.11	0.18*
IL− 27	0.52**	0.13	0.30**	0.59**	0.44**	0.51**

Not all data were available in all patients. qMalPCR, quantitative PCR of falciparum malaria in plasma; severity, disease severity according to WHO classification, see Methods; eGFR, estimated glomerular filtration rate; WBC, total white blood cell counts; vWF, von Willebrand factor. *Correlation is significant at the 0.05 level (2-tailed). **Correlation is significant at the 0.01 level (2-tailed)

### Plasma levels of IL-18 and IL-18bp during follow-up

**Fig. 3:**
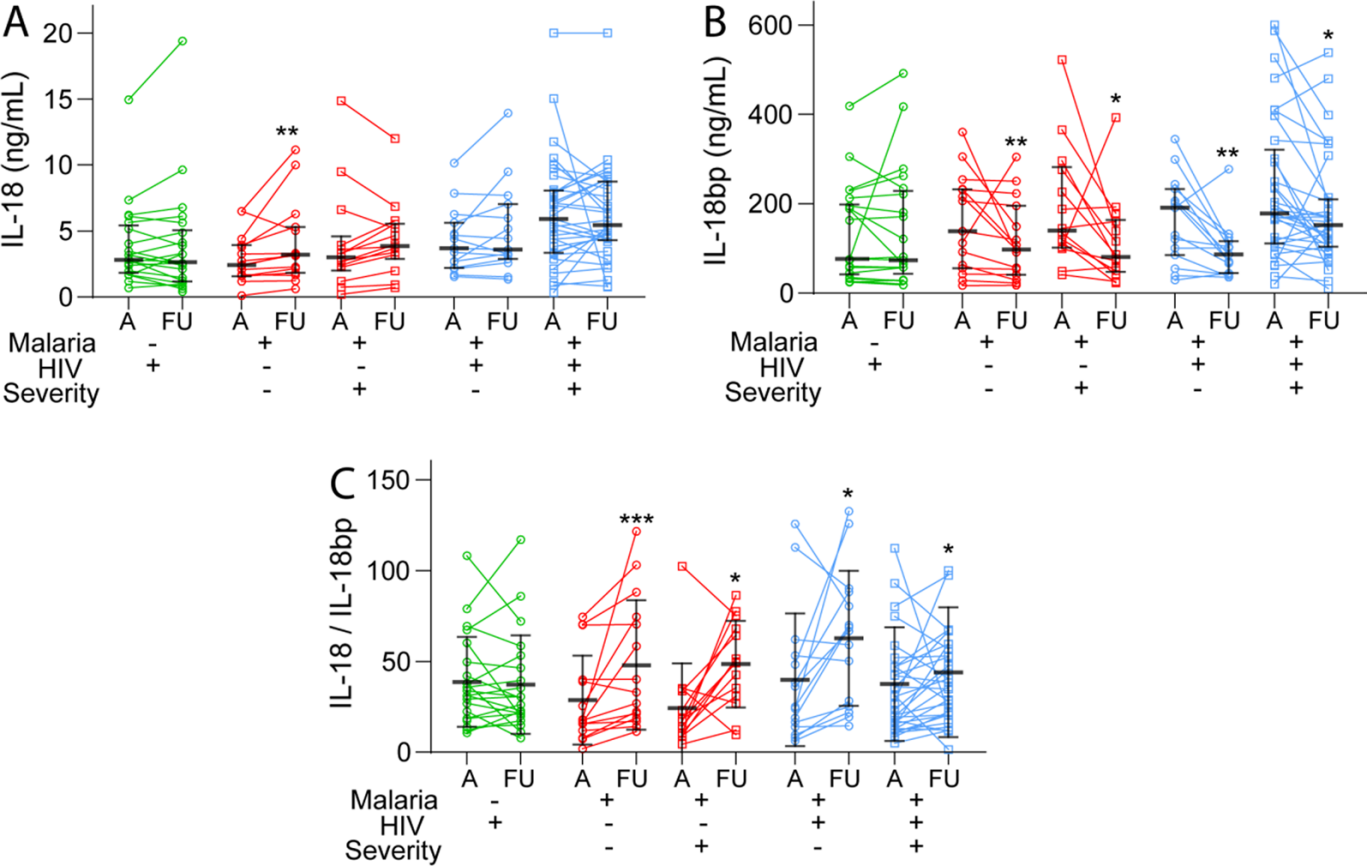
Plasma levels of IL-18 (**A**), IL-18bp (**B**) and the **C** IL-18/IL-18bp ratio during follow-up. Plasma levels of IL-18 and IL-18bp at admission and follow-up (FU, 48 h after admission) were available in 22 patients with HIV infection without malaria and in patients with falciparum malaria without (n = 29, of which 14 with severe disease) and with HIV infection (HIV + M, n = 48 of which 33 with severe disease). Lines in the scatter plot represent the median and 25–75th percentiles. *p < 0.05, ***p < 0.001 versus levels at admission

In 77 patients (HIV without malaria [n = 49], malaria only [n = 6], malaria and HIV [n = 22]) we also had follow-up samples taken in the hospital 48 h after admission (Fig. 3). Whereas there were no changes in plasma IL-18, IL-18bp and IL-18/IL-18bp ratio during follow-up in HIV-infected patients without malaria, the malaria patients showed a marked significant increase in IL-18/IL-18bp ratio independent of co-infection with HIV and disease severity (Fig. 3). This pattern seemed primarily to reflect a decrease in IL-18bp in all sub-groups of patients, whereas IL-18 did not change in patients with malaria and HIV but actually increased in patients with malaria only. Finally, during follow-up 10 patients with falciparum malaria of which 9 were co-infected with HIV died (in-hospital mortality), and these 10 non-survivors had significantly higher IL-18 levels than the 121 survivors (median [25th, 75th percentile] 7.5 [3.4, 10.1] vs. 3.3 [2.0, 5.5] p = 0.016). In contrast to IL-18, high levels of IL-18bp or IL-18/IL-18bp were not associated with in-hospital mortality.

### Effects of hemozoin and rhIL-27 on IL-18 and IL-18bp release from endothelial cells

**Fig. 4:**
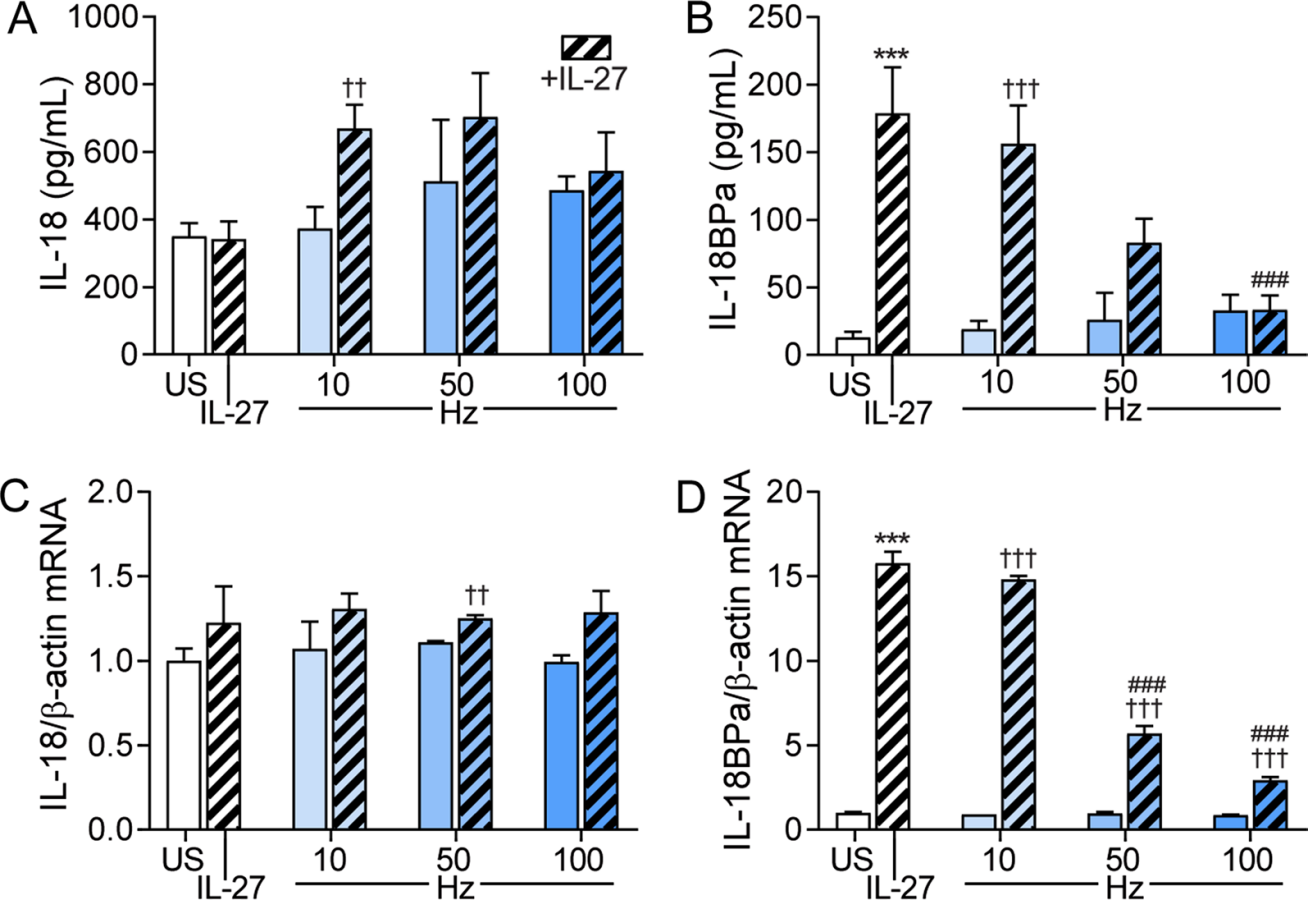
Effects of IL-27 on IL-18 and IL-18bp gene expression and release from hemozoin-exposed endothelial cells. Human aortic endothelial cells were primed with recombinant (rh)IL-27 (100 ng/mL, 1 h) and incubated with 10, 50 and 100 μg/mL hemozoin (Hz) for 22 h. IL-18 (**A**) and IL-18bp (**B**) were measured in supernatants from the cells with EIA. Gene expression analyses for IL-18 (**C**) and IL-18bp (**D**) were analyzed by qPCR, related to reference gene β-actin/TaqMan reference probes and normalized to unstimulated cells (US). Results are representatives of minimum three experiments and data are presented as mean and SEM. ***p < 0.001 versus US (white bar), ^††^p < 0.01 and ^†††^p < 0.001 versus Hz (blue bars) and ^###^p < 0.001 versus IL-27 (hatched bars)

We have recently shown that IL-27 may promote NLRP3 activation, an important source of IL-18 [13]. IL-27 has recently also been shown to increase IL-18bp in skin resident cells and in human ovarian cancer cells [15, 16]. However, no data exist on the effect of IL-27 on IL-18bp in endothelial cells that are more relevant for malaria infection. We recently demonstrated increased plasma IL-27 in this study population, positively correlated with degree of parasitemia [12], and as shown in Table 2, IL-27 was significantly correlated with IL-18bp in both malaria groups, and in malaria patients without HIV, IL-27 was also correlated with IL-18. We therefore next examined the ability of IL-27 to modulate the release of IL-18 and IL-18bp from endothelial cells with and without hemozoin exposure. Whereas hemozoin stimulation alone did not induce any release of IL-18 or IL-18bp in endothelial cells, hemozoin in combination with IL-27 induced a modest increase in IL-18 levels, being statistically significant at the lowest hemozoin concentration (Fig. 4A). However, the effect of IL-27 was rather modest, in particular at the mRNA levels, with a potential additive and not synergistic effects of IL-27 on the IL-18 release when given together with the lowest hemozoin concentration. Moreover, IL-27 markedly promoted release of IL-18bp from endothelial cells and interestingly, the stimulating effect of IL-27 on IL-18bp release was markedly and dose dependently attenuated when co-incubated with hemozoin (Fig. 4B). A similar pattern was seen at the mRNA level (Fig. 4C, D).

## Discussion

Several inflammatory mediators participate in the host response to malaria [1, 2], and in the present study we show a marked regulation of IL-18 and its binding protein IL-18bp during falciparum malaria, with particular high levels in patients with severe malaria disease that were co-infected with HIV. Moreover, during follow-up all malaria patients showed an increase in IL-18/IL-18bp ratio, irrespective of disease severity and co-infection with HIV primarily reflecting a decline in IL-18bp, suggesting a net inflammatory temporal effect in the IL-18 system. Finally, previous studies have shown that IFN-γ induce an up-regulation of IL-18bp as a potential counteracting mechanism [3]. Herein we show that IL-27, another cytokine with relation to T cell activation [12], markedly induced a release of IL-18bp from endothelial cells in vitro, and notably, this presumably anti-inflammatory effect was counteracted by hemozoin. Our findings suggest that the IL-18 system could be an important mediator in the immunopathogenesis during falciparum malaria, potentially also representing a target for therapy.

Based on studies in experimental malaria it has been suggested that IL-18 could have a protective effect in the infected host, at least partly related to its effect on NK cells [5, 22]. Moreover, a study in Papua New Guinea, suggests that IL-18 decrease the risk of clinical malaria in children through a modulatory effect on T cells [23]. Further, clinical studies in children have shown higher IL-18 levels in uncomplicated compared with severe falciparum malaria [24]. There are also some reports on adult falciparum malaria patients having raised IL-18 levels; Whereas some studies have restricted the inclusion to uncomplicated malaria showing elevated IL-18 levels compared to controls [8], some report no differences between uncomplicated and severe malaria, while others, on the contrary show the highest IL-18 levels in severe malaria [6, 7, 25]. However, the number of patients included in these studies was relatively low and none of the studies focused on co-infection with HIV. In the present study that included 131 patients with falciparum malaria, we found markedly raised levels of IL-18 in these patients compared with healthy controls with the highest levels in those co-infected with HIV and severe malaria disease. Indeed, malaria patients without HIV had similar IL-18 level as HIV-infected patients with similar febrile symptoms but without falciparum infection, illustrating the “inflammatory” contribution of HIV. Moreover, IL-18 levels at inclusion were significantly associated with disease severity and the degree of parasitemia, as assessed by qPCR. This pattern may contrast to some studies in children and it is possible that the effect of IL-18 in *P. falciparum* infection may differ between adults and children.

Like several other inflammatory cytokines, IL-18 has its own endogenous antagonist, IL-18bp that effectively forms a 1:1 high affinity complex with IL-18, exhibiting a very low dissociation rate [3]. High levels of IL-18bp have been reported in various inflammatory conditions like septicemia and various autoimmune disorders [3]. Previously, increased levels of IL-18bp, together with a wide range of other inflammatory markers, have been associated with falciparum malaria in pregnancy in a large cohort of HIV negative women from Malawi [11]. However, the present study is the first report of IL-18bp in clinical falciparum malaria in non-pregnant adults showing a significant association with disease severity and parasitemia. Moreover, whereas there were no changes (malaria patients co-infected with HIV) or a modest increase (malaria only) in IL-18 levels during follow-up, IL-18bp levels markedly decreased in both malaria groups, potentially reflecting a marked shift to inflammation within the IL-18 system during hospitalization. Indeed, during follow-up there was a rise in IL-18/IL-18bp ratio in all subgroups of malaria patients. The clinical importance of this pattern is at present not clear, and importantly, by binding the anti-inflammatory cytokine IL-37 [3, 26, 27], IL-18bp could potentially also have inflammatory effects.

IL-18bp does not correspond to the extracellular ligand binding domain of the IL-18 receptor and is not released from the cells by protease activity but is rather encoded by a separate gene [3]. The regulation of IL-18bp is not fully characterized, but IFNγ has been shown to increase IL-18bp levels, potentially as a counteracting mechanism to balance the inflammatory interaction between macrophages and T cells [3, 27]. In the present study, we show that IL-27 also markedly enhanced IL-18bp in endothelial cells both at the protein and mRNA level. We have recently shown increased IL-27 levels in this cohort of malaria patients potentially mediating both inflammatory and anti-inflammatory effects [12] and our data in the present study suggest that IL-27 could balance the activity of the IL-18 system by increasing the level of IL-18bp. Notably, at least in vitro, this anti-inflammatory effect was markedly attenuated by hemozoin, particular at high concentrations. If this effect is also seen in vivo, it could reflect an inflammatory effect of falciparum-derived hemozoin in the interaction between the parasite and endothelial cells, potentially mediating harmful effects on the host.

The present study has some limitations, since the follow-up samples during hospitalization were only obtained in a subgroup of patients. Moreover, correlation data do not necessarily mean any causal relationship, and the clinical consequences of altered IL-18bp is still not clear. Also, the lack of data CD4 + T cell counts on most of the HIV-infected patients may limit the interpretation of the data from these patients. Finally, comparative data from patients with other types of malaria would have strengthened our study.

## Conclusion

Our data add the IL-18/IL-18bp axis to the inflammatory pathways that are dysregulated during falciparum malaria. Based on the availability of IL-18bp as a treatment option in clinical trials [28], our data may suggest that this therapeutic approach also could be investigated in falciparum malaria.

## Data Availability

The datasets used and/or analyzed during the current study are available from the corresponding author on reasonable request.

## Abbreviations

HAoECs: Human Aortic Endothelial cells
IL: Interleukin
IL-18bp: IL-18 binding protein
IFN-γ: Interferon-γ
NK cells: Natural killer cells
*P. falciparum*: *Plasmodium falciparum*
(q)PCR: quantitative PCR
vWF: Von Willebrand factor
